# Barriers and facilitators for access and utilization of reproductive and sexual health services among Female Sex Workers in urban and rural Maharashtra, India

**DOI:** 10.3389/fpubh.2022.1030914

**Published:** 2022-12-08

**Authors:** Suhas Shewale, Seema Sahay

**Affiliations:** ^1^Division of Social and Behavioural Research, Indian Council of Medical Research-National AIDS Research Institute (ICMR-NARI), Pune, India; ^2^Interdisciplinary School of Health Sciences, Savitribai Phule Pune University, Pune, India

**Keywords:** Female Sex Workers (FSWs), Reproductive and Sexual Health (RSH), unintended pregnancy, abortion, healthcare service utilization, condom usage, qualitative research, India

## Abstract

**Background:**

The public health interventions among Female Sex Workers (FSWs) have mainly addressed HIV/ STI prevention. The focus of the HIV prevention program on FSWs' Reproductive and Sexual Health (RSH) has been limited, thus, rendering them at a higher risk of unintended pregnancies, delayed pregnancy detection, and utilizing unsafe abortion methods.

**Methods:**

A multistakeholder analysis was performed to study access and use of RSH services among FSWs in urban and rural India. Between January 2016 and June 2019, a qualitative grounded theory approach was used to explore the FSWs' perspectives and experiences about services pertaining to HIV prevention, Antenatal Care (ANC), child delivery, abortion, and pregnancy prevention. Using purposive and convenience sampling, 29 In-Depth Interviews (IDIs), 2 Focus Group Discussions (FGDs) and 22 Key Informant Interviews (KIIs) were conducted with consenting FSWs and indirect stakeholders, respectively. Verbatim translated data was entered in NVivo12 Software and analyzed inductively.

**Results:**

The following themes emerged: (1) Condomless sex, unintended pregnancy, vertical transmission, (2) Signs/ indication used for pregnancy detection causing delay (3) Pregnancy prevention methods used, (4) Pregnancy prevention or AIDS prevention, (5) Legal formalities as a barrier to access RSH, (6) Differential facility preference.

**Conclusion:**

Pregnancy prevention is a greater motivation for condom use than HIV prevention among FSWs. Therefore, there is an emerging need to reallocate public health resources and redesign policies to meet the RSH needs of FSWs, especially for the prevention of unintended pregnancies. FSW-focused Information Education Communication (IEC) strategies for RSH service utilization are essential to reduce the burden of unintended pregnancies. The National HIV Targeted Intervention (TI) program needs to include pregnancy testing services and information to non-barrier contraceptive methods. An ambient policy environment calls for examining the need for male involvement in pregnancy, family planning and abortion decisions.

## Introduction

Female Sex Workers (FSWs) are disproportionately affected by HIV globally and experience systemic barriers to accessing HIV prevention and treatment services (1–4). Stigma, criminalization of sex work, sexual violence, discrimination at health facilities due to their occupation, and disadvantaged socioeconomic status pose barriers for FSWs to access health care services (5–9).

FSWs experience a high burden of unintended pregnancies within complex relationships with clients and non-paying sexual partners and report limited uptake of family planning methods (10–13). Condoms are often relied upon as the sole method of contraception among FSWs (14, 15). Data on the use of dual methods for contraception in the context of using condoms with regular partners or spouses in India is limited. Forty percent of the FSWs in Karnataka (16) and 26% in Goa (17) states reported using condoms as the only contraceptive.

Low contraceptive use and the high burden of unintended pregnancy affect a large number of women in Low-Income and Middle-Income Countries (LMICs) and result in poor reproductive outcomes for maternal and child health and preventable mother-to-child HIV transmission risks (10, 18, 19). Pregnancy incidence in FSWs across 25 studies ranged from 7.2 to 59.6 per 100 person-years (18). Of the 946 FSWs surveyed in Zambia, 84.1% reported being pregnant at least once, 61.6% reported an unplanned pregnancy, and 47.7% FSWs had one terminated pregnancy (20). Another study from Côte d'Ivoire reported that 64% of the FSWs had more than one termination of pregnancy, of which half were not medically supervised (10). In India, among 260 FSWs surveyed in Goa for their contraception use, 26% had undergone at least one abortion (17).

The coercive condomless sex among FSWs is often the cause of unintended pregnancies (11, 21, 22). Adverse pregnancy outcomes have been reported among FSWs who faced sexual violence (23), which resulted in sequalae of delay in pregnancy diagnosis, HIV tests and ART initiation, increasing the risk of vertical HIV transmission (24). According to the WHO report, in the South-East Asian region, India has the highest burden of new pediatric HIV infections (25). HIV prevalence among ANC clinic attendees in India was 0.24% in 2019 (26). A systematic review reported the pooled HIV prevalence of mother to child transmission in India to be 8.76% among babies born to HIV positive mothers (27). The data for vertical transmission of HIV from FSWs to their children is not available for India. Information related to the engagement of FSWs in antenatal care and during pregnancy is a critical gap observed among FSW mothers (16, 28).

In India, contraception services, including the promotion of condom as a way to prevent pregnancy among all women, is promoted through the Reproductive, Maternal, Child and Adolescent Health (RMNCH+A) (29) program. Other reproductive services such as antenatal care, delivery and safe abortion services are also included. However, condom promotion and marketing of condoms, HIV prevention and treatment of Sexually Transmitted Infections (STIs), community outreach and community system strengthening are key elements of Targeted Interventions (TI) for FSW (30). The common intervention across these programs is “condom use” focusing on two populations of “non-FSWs women” and “FSW women” for two different purposes, viz (I) pregnancy prevention among the general population in the RMNCH+A (29), and (II) HIV prevention for FSWs in the HIV Targeted Intervention program (30).

The research and public health interventions among FSWs seems to have focused on HIV/ STI prevention, reducing sex workers to potential vectors of HIV infection and ignoring their reproductive and sexual health needs. Therefore, a gap exists between FSWs' needs and preferences in using modern methods of contraception and their access to contraceptive services in India. We present an exploratory multistakeholder analysis of the barriers and facilitators for access and utilization of Reproductive and Sexual Health (RSH) services among FSWs in the urban and rural districts of high HIV prevalent state of Maharashtra, India.

## Methods

### Study design

A qualitative study was conducted among FSWs, and the stakeholders in four purposively selected rural and urban blocks of Maharashtra, India. These blocks belong to the Category A NACO (31) districts with more than 1% HIV prevalence among ANC attendees. We used grounded theory approach to conduct this study.

A total of 29 In-depth Interviews (IDI) were conducted with FSWs, and 22 Key Informant Interviews (KII) were conducted in this study. Two Focus Group Discussions (FGDs) were conducted with 8 FSW participants in each group at the rural and the urban study site, respectively. This study is reported using the COnsolidated criteria for REporting Qualitative research (COREQ) guidelines (32).

### Study population

The operational definition of FSW in this study is “Females, over 18 years of age, who receive money or goods in exchange for sexual services, either regularly or occasionally (33), and who may or may not consciously define those activities as income-generating (34).”

NACO's definition of typology (35) is used as follows: Street based-FSWs who solicit the clients on the street or in public places such as parks, railway stations, bus stands, markets, and cinema hall; Brothel-based-FSWs soliciting clients at brothels; Phone-based or FSWs at workplace (36): sex workers who solicited their clients on phones or at the workplace. FSWs who were 18–49 years of age and self-identifying as sex workers participated in the IDI.

The Key Informants included representatives from the Community-based Organizations (CBOs), Non-Governmental Organizations (NGOs), brothel owners, health care providers from private, public health facilities, alternative medicine doctors, doctors from CBOs/ NGOs, outreach workers and program personnel.

### Recruitment of study participants

Maximum variation purposive sampling technique (37) was employed to enroll participants with varied experiences or perceptions about pregnancy, access to antenatal care, abortion care across different categories, a wide range of age groups, and rural and urban study areas. The data collection followed an inductive approach (38, 39) to recruit participants based on the descriptive needs of the emerging concepts and theory (37). The data collection continued until theoretical saturation was reached.

a. Recruitment of Key Informants: Twenty-two key informants were recruited for the study: Brothel owners (*n* = 3), Counselors from ART/ Integrated Counseling and Testing Centers (*n* = 3), Doctors from Govt. Health Facilities, gynecologists, medical officers at ART centers (*n* = 4), Private Healthcare Providers, alternative medicine practitioners (*n* = 4), Outreach workers (*n* = 3), CBO representatives (*n* = 3), and Program personnel (*n* = 1). The snowball technique was used for recruitment. The key informants were contacted either by telephone, email, or personally, purpose of the study was explained and face-to-face interviews were conducted.

b. Recruitment of FSWs: A total of 29 IDIs were conducted among FSWs at urban (*n* = 17) and rural sites (*n* = 12) respectively. The typologies were as follows: brothel-based (*n* = 14), phone-based (hidden, privately operating) (*n* = 9), street-based (*n* = 4) and FSWs soliciting clients at workplace (*n* = 2).

Strategies used for recruitment of FSWs were as follows:

I. CBO/ NGO/ Community engagement: To identify street/ phone-based FSWS, the investigator was guided by the local grocery shop owners, tea stall owners, brothel owners, auto-rickshaw drivers, lodge owners, *paan* (betel leaf) *shopkeepers* and healthcare providers. Potential participants were approached through local peer educators from 6 local CBOs and NGOs.
II. Snowball: Street-based FSWs operating on the phone and hidden FSWs were referred by the participant.
III. Observation and recruitment: Women were observed soliciting clients at the particular fixed location identified by the investigator. They were approached, rapport was established, and FSWs from hidden brothels were contacted directly by this method.

### Study tools

The data was collected using pilot tested interview topic guides and an FGD guide.

a) Topics in the IDI guide comprised reproductive and sexual health, antenatal care, contraception, pregnancies, unintended pregnancies, abortions, understanding of the vertical transmission of HIV, practices to prevent vertical transmission, knowledge of existing RSH facilities, decision making and influencers on care-seeking, preferences, and experiences of engagement in RSH care services. The topics were structured to build rapport with the respondents, with more emotive questions being asked later in the interview.
b) The KII guide focused on the engagement of FSWs in RSH care, influencers for service access and utilization, existing gaps in public health facilities and policies, and capacity and infrastructure building needs.
c) The focus of the FGD guide was: factors that influenced decision making to access RSH facilities, social norms, FSWs' views/ experiences on pregnancies, pregnancy prevention, abortions, social and emotional experiences with RSH facilities, and community expectations.

### Data collection

Between January 2016 and June 2019, data was collected by a trained female doctoral student who did participant observation for 30 weeks prior to the initiation. She was introduced as a researcher interested in social and behavioral research and working with HIV key populations. IDIs were conducted in a safe and private place and at a time that was agreeable to the study participants at CBO/ NGO offices, brothels, hotel rooms, and community clinic facilities run by ICMR-NARI. The average interview time with FSWs was 50 minutes. Two interviews lasted for only 10 minutes because the participants left the interview on the pretext and did not return. The data has been included in the analysis. KIIs were conducted at the participant's offices, CBO offices, brothels, or at their place of residence and at a time convenient to the participants. KIIs lasted for 45 to 60 minutes.

Interviews and FGD were audio-recorded using an electronic recorder (SONY ICD-PX470) with the participant's consent and, field notes were also taken. Permission was not accorded for the audio recording of 01 FGD and 02 IDIs for which handwritten notes were taken. Interviews were conducted in the participants' preferred language (English/ Hindi/ Marathi). During data collection, data was analyzed simultaneously, which helped evolve the study guides and informed the participant selection. Repeat interviews were conducted with 2 key informants and one IDI participant for the missing information (Box 1).

**Box 1:** Focus of the repeat interviews.

**Content analysis finding**	**Repeat interview focus**	**Relevant quotes**
**Repeat interview (*****n*** **=** **3)**		
Missing information related to FSWs reversing the sterilization procedure	Exploring the reason why is this procedure was reversed	“*I am going to =xyz= and open my pipe [/tubal ligation/]. Will you give birth to his child and again if he got married [/to someone else/] and didn't keep the relation with you? I am going to reopen…when he gives his entire estate in my name…baby's name. I can easily live on his estate”* (IDI_U_03, privately operating FSW, urban)
An important component of the program is providing contraception counseling outreach services, missing information in the interviews	To understand how are the contraception counseling services provided to FSWs in the field	“*do not come to the brothel to talk about pregnancy, sisters [/nurse, visit/] for HIV camp”* (KII_17, brothel owner) “*Outreach workers do not go there, mostly, other activities, they have their own peer educators to tell”* (KII_14, program personnel)

### Data management and analysis

The audio-recorded interviews uploaded on GoldWave Inc software were transcribed and translated verbatim. The grounded theory approach was employed to generate new and emerging inductive codes from the data. Using the constant comparison method, codes were merged into similar categories, core concepts were identified. Transcripts were read iteratively by SSh and SSa to identify recurrent themes to search for meanings and patterns. Emerging themes and new codes were identified inductively from the data. Data was analyzed using NVivo 12 software.

### Ethical considerations

The study was approved by the Institutional Ethics Committee of ICMR-National AIDS Research Institute, Pune, India and the Ethics Committees of the partner CBOs and NGOs. Written informed consent was obtained from all participants prior to conducting and audio recording the interview. A reimbursement of INR 150 (1.9 USD) was provided. Fourteen participants were not reimbursed as mandated by the CBOs or NGOs. The ethics committees were informed. Participants were informed prior to conducting the interviews.

## Results

Of the 27 key informants approached for the interview, interviews were conducted with 22 key informants. One key informant was not permitted by their CBO and 4 key informants initially agreed to participate, but finally did not participate on repeated contacts. Based on the initial content analysis, 3 repeat interviews were conducted with 2 key informants and one IDI participants. These repeat interviews lasted for around 10–15 minutes. Few additional items were explored with these participants (Box 1).

Of the 46 FSWs approached, 29 IDIs were conducted. Of the 17 failed recruitments, 7 agreed but were never reachable afterwards, 4 FSWs were not eligible (>49 years), 2 could not respond in Hindi or Marathi, and 4 did not have time. Two IDIs were interrupted as the respondent went to attend to clients. One FSW felt discomfited and did not complete the interview. Incomplete interview data were included in the analysis.

The median age for the FSWs was 32 years (Range: 21–49) (Table 1). More than 40% of the FSWs were between 26 and 45 years of age. Seven (24.1%) FSWs reported unmarried status. Others were either married (31%) or were divorced/ separated/ widowed (37.9%). Of the 29 FSWs, 3 (10.3%) were menopausal and 12 (41.3%) were HIV positive. Two third of the FSWs had undergone at least one abortion in their lifetime, and two were planning to terminate the pregnancy at the time of the interview. All FSWs reported using condoms. Nine (31%) had undergone a permanent method of pregnancy prevention. These 9 women were either married, divorced or widowed. Ever using an intrauterine device (IUD) and oral contraceptive pills were reported by 4 (13.7%) and 5 (17.2%) participants respectively.

**Table 1:** Demographic characteristics of FSW participants.

**Characteristics**	**Frequency (%)**
**Age**	
< 25	8 (27.5)
26–45	15 (41.7)
>45	6 (20.6)
**Education**	
Functional literacy	13 (44.8)
Secondary/higher education	6 (20.6)
Primary education	5 (17.2)
Cannot read, write, sign	16 (55.1)
**Marital status**	
Married	9 (31)
Unmarried	7 (24.1)
Separated/divorced/widowed	11 (37.9)
**No of pregnancies**	
0	1 (3.4)
1	3 (10.3)
2	9 (31)
3	6 (20.6)
4+	8 (27.5)
Missing data^*^	2 (6.8)
**No. of abortions reported**	
0	6 (20.6)
1	11 (37.9)
2	6 (20.6)
3+	3 (10.3)
Missing data^a^	3 (10.3)
**Ever reported using non-barrier method for pregnancy prevention**	
IUD	4 (13.7)
Oral contraceptive pills	5 (17.2)
Emergency contraceptive pills	2 (6.8)
Permanent method of contraception	9 (31)

* Missing data due to participants leaving after the consent procedure or refusing to answer.

**Figure 1:**
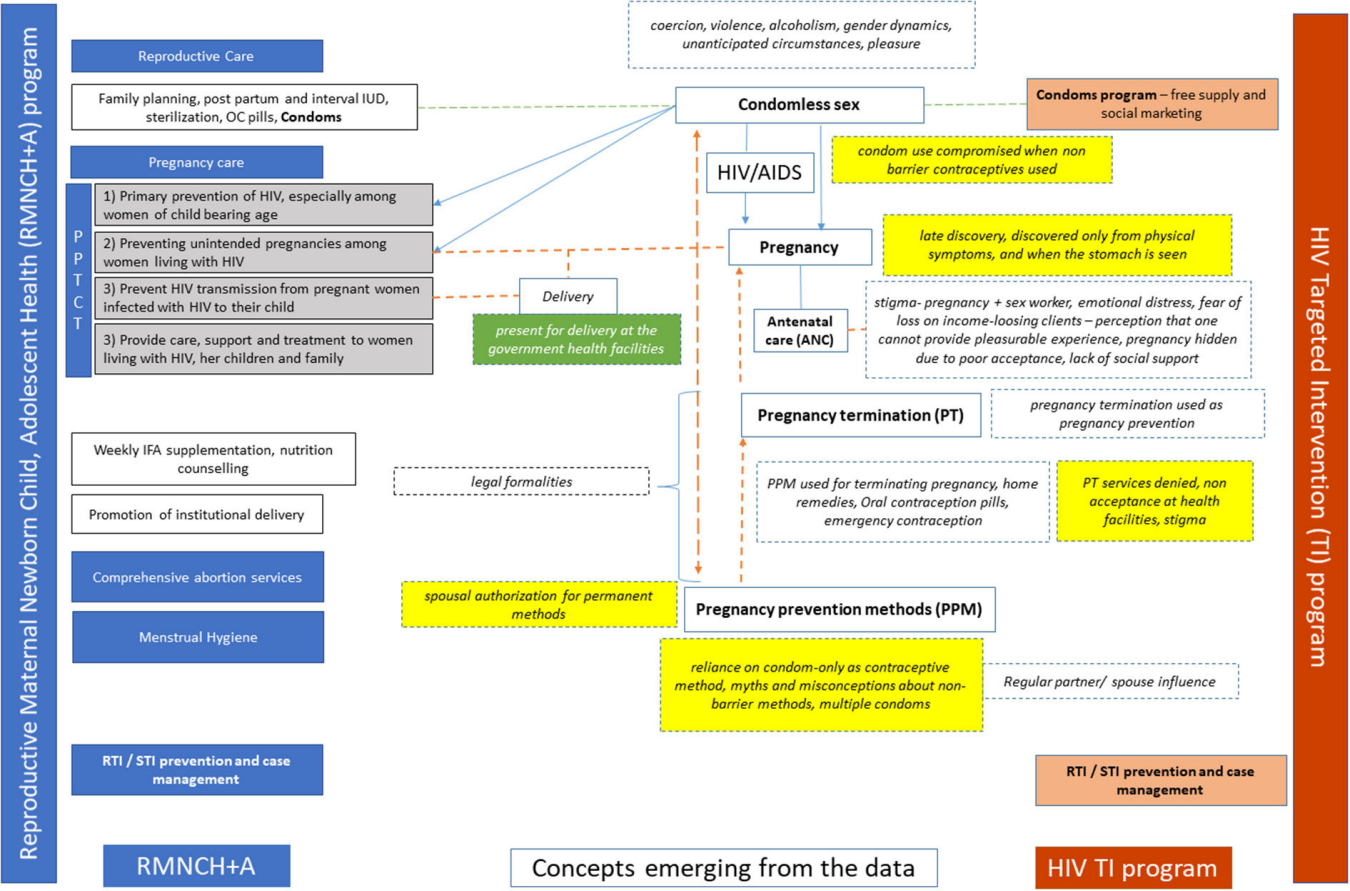
Analytical framework highlighting gaps and opportunities for FSWs' engagement in reproductive health services within the existing RMNCH+A and the Targeted Intervention programs.

The qualitative data highlights the barriers and facilitators for FSWs' engagement within the RSH care services in the context of HIV Targeted Intervention program (Red color) and the RMNCH+A (Blue color) program in India in Figure 1. The analytical framework highlights the issues of unintended pregnancies, delayed pregnancy detection using locally known signs and indications, and myths around the long durations of missed menstrual cycles, which affects the FSWs' engagement in antenatal care and services for safe abortion of unintended pregnancies. Unplanned sexual encounters with clients or regular partners resulted in condomless sex. Despite experiences of unintended pregnancies and unplanned condomless sexual encounters with clients and spouse, the dual methods of contraception were considered futile. There were myths and misconceptions around non-barrier methods of contraception. There was reliance on the “condom-only” as a pregnancy prevention method. Further, sexual violence by a spouse and regular partners for using contraception limited the use and demand for non-barrier contraceptives. The analytical framework highlights the environment at healthcare facilities being non-ambient in terms of access to contraceptives, fear of being asked for identifying/ sexual partners' information, and restrictive policy environment which further limits the access. Continuing with unintended pregnancies and delayed access to antenatal care, knowingly or unknowingly, leads to poor access to HIV Prevention of Parent to Child Transmission (PPTCT) care services. All these pose an additional risk of vertical transmission of HIV.

The emerging themes are underscored by gender inequity, power issues, stigma, and discrimination. Participants discuss how sex work, violence, and discrimination faced by the FSWs are often areas of antagonism between themselves and people holding power, viz. their clients, regular partners, brothel owners, spouse and law administrators. Internalized stigma due to their negative experiences of being in this occupation plays a critical barrier in accessing RSH services among FSWs. The emerging themes are delineated and described as follows:

- *Condomless sex, unintended pregnancy, vertical transmission*
- *Signs/ indication used for pregnancy detection causing delay*
- *Pregnancy prevention methods used*
- *Pregnancy prevention or AIDS prevention*
- *Legal formalities as a barrier to access RSH*
- *Differential facility preference*

### Condomless sex, unintended pregnancy, vertical transmission

Consistent condom use and safe sex behaviors are promoted among FSWs for HIV prevention in the targeted intervention programs. All FSWs felt sex without a condom was perceived as a source of acquiring HIV/ STI and getting pregnant. All women reported using condoms with clients for HIV/ STI prevention because “*nobody can be trusted*.” However, in practice, FSWs, both at the rural and the urban sites, described encounters when “*the husband did not listen*,” the condom being “*ruptured*” because the “*customer does not sit well*,” and “*the client deliberately tore the condom*.” The targeted intervention program's focus on addressing accidental pregnancies among FSWs emerges as a gap.

Street-based FSWs have safety issues, and ensuing condomless and coercive sex lead to a higher risk of unintended pregnancies. An HIV counselor a stated:

*“Suppose a truck driver comes, the truck driver stops the vehicle and the woman proposes to him. Both of them agree for the business. Along with the driver, cleaner and owner of the truck may also be there in the vehicle. All three of them will have sex with her. Such relations without condom may result in pregnancy, STI, RTI because naturally, people, who are having sex forcibly, are not going to use condoms.”* (KII_08, Counselor).

A sex worker at the rural site described an event of non-consensual sex with multiple clients.

*“One man came in an auto-rickshaw. He said that there were 5–6 more people waiting there. All of them came from both sides of the auto and forced me to sit [/have sex/] there…took the autorickshaw to a pit/ ditch at* = *Name of place* =*. Yes, they took me there and told, come on sit [/have sex/] now.”* (IDI_R_06, hidden FSW, rural).

Women shared not using condoms with “*someone who loves*.” The gender dynamics and the power that the “husband” holds in the family renders the woman less able to control condom use. A brothel owner shared:

*“They [/spouse/] beat, about sex. They [/girls/] let themselves to be used the way husband wants, they let him have sex.”* (KII_17, brothel owner).

“*Someone [/spouse or client/] beats for sex. They [/girls/] let themselves to be used by the customer. These girls do anything; they let him have sex for love…It may be a customer or anyone else. Then they become pregnant. Some of them have to do it forcibly.”* (IDI_U_04, street-based FSW, urban).

Alcoholism in this occupation emerged as another cause of unintended condomless sex as an FSW shared:

*“[/He/] put on condom; condom didn't tear. Like this fast he sat [/had sex/], and that condom also had blood on it. So due to that it [/pregnancy/] may have occurred or due to something else, it occurred. I don't know. No, when I am in senses [/not under the influence of alcohol/], that time I understand whether it [/condom/] is torn or not.”* (IDI_R_09, street-based FSW, rural).

“*I had drunk too much [/alcohol/] so it [/condomless sex/] could have happened that time. I had insisted with the customer to use condom but may be, after I had got drunk, he hadn't used it. So, everything in their mind comes later. [She said] It is already agreed to do it [/sex/] with condom. [She voiced aloud] It will not happen purposely.”* (IDI_U_03, privately operating FSW, urban).

Four respondents reported using a female condom as covert method of contraception when men refused to wear condoms. However, access to female condoms was a challenge due to high cost and additionally, it did not fit well and slipped. A false sense of security emerged, showing complacency toward condomless sex as follows:

*“We use a condom mostly. Condom is good. One more thing is that if any man sits [/has sex/] without condom, we should put a finger and wash.”* (IDI_R_03, hidden FSW, rural).

Five women believed washing their bodies with disinfectants may prevent pregnancy if condoms are not used. This was reported to be the solution to avoid “disease” and pregnancy.

*“…make use of Dettol [/antiseptic, disinfectant/] in order to not have any disease and not to get pregnant. We used to wash it off with Dettol water.”* (IDI_U_05, street-based FSW, urban).

Condom availability was a challenge for FSWs at the rural sites. Condoms were not available at the CBO offices and FSWs reported being allowed to take only a limited number of condoms from the government health facilities.

*“In the hospitals, we should get condom properly [/enough quantity/], that's all. Take these many; take those many [/take only a few condoms/], only this much stock is available [/limited availability of condoms/], that much stock available*.” (IDI_R_01, hidden FSW, rural).

At the urban sites condoms were reported to be easily available at the CBO offices, government hospitals and also at the lodges by FSWs.

### Signs/indication used for pregnancy detection causing delay

Pregnancies were generally unwanted in this population.

*“This is because many males believe that having sex with a woman who has had many deliveries or abortions doesn't give much pleasure. That's why they [/FSWs/] feel that their pregnancy should not be revealed to any of their clients.”* (KII_08, counselor).

Pregnancies were accidental and “discovered” only from visible/ felt physical symptoms such as tiredness, sleepiness, nausea, and vomiting, but rarely due to changes in the menstrual cycle. FSWs from the rural and the urban sites mentioned that delayed or missed menstruation could be a sign of “*less blood*” in the body and “*weakness*.” Most women in the urban and rural settings realized about their pregnancy late only after the “stomach was visible” or after they had thought of all other options.

An FSW who discovered her pregnancy in the seventh month, shared:

*“I got periods after one and half years. Then I thought, I have no strength in my body. How would I know I will become pregnant? Found out about pregnancy in the seventh month. Even if 9 months [/of pregnancy/] would have crossed, my stomach is not seen. You won't see my stomach, regardless of anything my stomach will not be seen. It only looks like the stomach that comes out after eating meals.”* (IDI_2, FSW).

A pregnancy test was recommended as the last resort by the brothel owner to one of the girls after she missed her periods for several months.

*“She missed it [/periods/]. But what happened in her case is that, she had lot of bleeding due to piles. She said, “Mummy, I had a lot of bleeding, and because of that, there is no blood remaining in my body. That's why I did not have periods”…but…we are experienced. So, I thought, she had all the treatments, and everything is done; now only one test is remaining, which is this [/pregnancy test/].”* (KII_17, brothel owner).

The FSW profession stigmatizes pregnancy. She hides it from customers, and she has no family support. Such situations probably lead to procrastinating visits to antenatal care, as shared by a NGO representative:

*“…she goes to the last stage at the time of delivery. But in normal women [/women in the general population/], after two and a half to 3 months [/of pregnancy/], in-laws or husband, parents take her to the hospital. In case of FSWs, this relation is still not openly acceptable. So, at least, I feel, this is the reason she does not go early to the hospital. [/she goes/] very late…when her belly starts showing clearly that she is carrying a baby, in the seventh, eighth month…now, no more customers are going to come to me.”* (KII_07, NGO representative).

The situation of delayed antenatal care has grave implications for the woman's life. A healthcare provider shared his experience:

*“She was an FSW. She got married to a client and became pregnant. The husband had a criminal record. Husband was in jail; she never came [/during pregnancy for ANC/]. Came directly toward the end [/for delivery/]. She was having an abruption, profuse bleeding and was in shock. She was strong…died.”* (KII_09, healthcare provider).

Unintended pregnancies, consequent delay in accessing antenatal care and vertical transmissions go together in the case of FSWs. Delay in pregnancy detection had implications for prevention of vertical transmission. In our study some FSWs were HIV infected who had late pregnancy detection and missed early opportunity of prevention of vertical transmission. An FSW shared about delay in pregnancy detection leading to vertical transmission:

*“I did not want to continue with the pregnancy. At that time, I wanted to abort it. But the doctor said that you cannot abort [/terminate/] it. It was 7 months, so I kept… whatever happens, is in God's hands. One child does not have it, but my other child has [/HIV infection/].”* (IDI_2, FSW).

### Pregnancy prevention methods used

Prevalent myths, misconceptions and cultural beliefs about pregnancy prevention methods were rampant. One of the respondents felt that she was not welcome at the medical termination of pregnancy service facility, and therefore, she attempted pregnancy termination with contraceptive pill:

*“I didn't want a baby then. They did not let me do the abortion or did not even take my name [/register my name, admit/]. I didn't get medicine [/for terminating pregnancy/]. That time, I bought* = *Mala D* = *[/oral contraceptive pill/] from a medical store. I had the pill…but the problem [/periods/] did not come.”* (IDI_R_10, street-based FSW, rural).

“* [I am] 3 months pregnant. But I took 72-h pill [/emergency contraception/]. If it [/abortion/] does not happen, I will see the doctor next month. People like you [/non-FSW, not drunk/] can go [/to seek pregnancy termination services/]; not me.”* (IDI_R_09, street-based FSW, rural).

Due to the fear of condoms slipping or breaking, four women out of twenty-nine suggested using more than one condom while having sex. Incorrect usage of condoms and myths about taking a bath after condomless sex was common among the study respondents.

*“You don't get pregnant if you use a condom. Suppose you are my customer, and if I have sex with you, I will use two condoms. If only one condom is used it may leak. If two [/condoms are used/], the liquid [/semen/] will remain there [/inside the condom/]; it will not come in contact with me. So, pregnancy is prevented [/if two condoms are used/]. If it [/semen/] comes in contact with my body, then only I will be pregnant. Who will take care of the future then? So, what we do is, we take a bath with hot water after having sex with the customer.”* (IDI_U_14, hidden FSW, urban).

Both FSWs from the rural and the urban sites did not want to use oral contraceptive pills and IUD. Women had many misgivings about non-barrier methods of contraception. Common specific harms were voiced about the oral contraceptive pills, such as “*hitting the kidneys*” and “*affecting the sac*.” Women also shared their concerns about the oral contraceptive pills “*clearing the uterus and then causing quicker pregnancy in case of condomless sex*.”

*“My sister could not conceive because of that [/oral contraceptive pills/]. However, due to these tablets, she had to get her sac of child [/uterus/] removed; it had completely got rotten and had decayed and become like a dough…it had become piece-piece [/broken into pieces/] because of the tablets [/oral contraceptive pills/].”* (IDI_R_03, privately operating FSW, rural).

Participants shared their perceptions that the IUD—Copper T may rust inside the body and cause “*cancer*” or that it may “*travel to the heart*.” Customers could feel it.

*“…causes problems or pricks and customer complains” (11 / 29 FSWs)*.

### Pregnancy prevention or AIDS prevention

The only concern the study participants had was not to become pregnant because it interfered with their business. Pleasing and satisfying clients was over and above anything, even at the cost of acquiring HIV infection by not using condoms. Nuances of behavioral disinhibition emerged:

*“Once Copper-T is fixed, then we can have sex without condoms. You will not get pregnant, because it prevents pregnancy…Yes, one can do it without using condoms… You can have AIDS, other diseases, but some people have relations with specific women, and as per their wish they decide whether to have sex with a condom or without it. If someone pays you good money[/for condomless sex/], then many women can't say no to them.”* (IDI_U_14, brothel-based FSW, urban).

Preference for condoms emerged as they could prevent both HIV and pregnancy, and there is no need to use any other method:

*“All the other methods there, no one uses them mostly. Only condom is used more, as condom is used there… Family planning is automatically done there.”* (KII_18, healthcare provider).

“*People here use condoms only, there is no need of* =*Mala D*= *[/contraceptive/] tablet. Now we don't need any of the other methods [of pregnancy prevention], [/condoms are/] good for everything…there is no need of pills if you use condoms.”* (FGD respondent, rural).

Even though condoms are the preferred barrier method, frequent events of condomless sex are reported, either coercive or by choice, to earn more money or please their partner/ client. Therefore, need for other methods of pregnancy prevention emerges. For example, an FSW who had experienced an unintended pregnancy in the past demonstrated her flippant attitude toward condom use when she was asked about not using condoms with regular partner:

If there is a regular relationship, FSWs are not empowered enough to go against partners' wishes.

*“Nothing, the regular partner says no [for condom/]”* (IDI_R_11, brothel-based FSW, rural).

“*My husband used to scold me because he didn't want me to take pills [/oral contraception/]. So, like this, I missed pills and became pregnant. It didn't get aborted by eating all these things. I continued the pills, and still, it didn't get aborted. Even after eating papaya, it didn't get aborted. Then came to the hospital. But it continued. The baby was born after all.”* (IDI_R_02, privately operating FSW, rural).

We did not come across any major concern about HIV prevention among study participants. However, a desire for motherhood emerged, which might affect condom use and pose the risk of HIV acquisition.

### Legal formalities pose a barrier to accessing abortion/ family planning services

Hesitancy and fear among FSWs in accessing antenatal care and Medical Termination of Pregnancy (MTP) services emerged due to legal paraphernalia required for MTP in India. An FSW who had 12 pregnancies in her lifetime shared:

*“How? [/how can I go?/] They used to say, go bring [/your/] husband. My children are all from the business [/sex work/]. Then? They ask who is the father. What will I tell them? You tell me? There are some things that cannot even be told…It is difficult, the person can hide once, once the person can tell with closed curtain [/secretly/], twice will do, but if I do it again and again, who will cooperate with us, tell me…will they do*?” (IDI_U_04, privately operating FSW rural).

A healthcare provider shares that women cannot access permanent methods of contraception unless they are legally married, which may pose a challenge for the FSWs:

*“And again, we can only recommend temporary methods to them. According to the Family Planning Act, we cannot do it on unmarried people.”* (KII_09, healthcare provider).

The health care providers do not support abortion which an FSW needs:

*“If you tell the doctor [/for abortion/], they will not write it down for you. They ask, why do you want to drop [/abort/], and they curse. I have received curse; that is why I am telling you.”* (IDI_U_06, brothel-based FSW, urban).

All this instills fear in the minds of the FSWs, who therefore try to hide their pregnancy status but still try to procure medicines that they believe would induce abortion:

*“You know, madam, if I want to get this medicine [/for abortion/] from there* =*XYZ*= *hospital, I tell I want medicine, I have missed my MC [/menstrual cycle/], and I wish to have regular monthly MC. Otherwise, if we go there as regular patient, getting such medicine after getting examined by them, is not allowed at that place. If ladies like you [/social workers/] are there inside, we explain to them. Madam, for 2–3 months, I haven't got my MC, what to do? So, she says, if you tell the doctor that way, they may not give you the medicine. Then she sends us after teaching us what to say. We take case paper inside, if you tell the person who gives the medicine that you are pregnant, they will not give you the tablet [/for regular periods/]. You just tell the doctor that you haven't got your MC, and take medicine whatever the doctor gives, and have the pills.”* (IDI_R_03, privately operating FSW, rural).

In both the urban and rural settings, FSWs reported about healthcare providers refusing to perform medical termination of pregnancy. They reported experiencing stigmatizing behaviors of healthcare providers if they sought abortion services. Therefore, FSWs did not want to access medical services for terminating the pregnancy, rampant use of locally prevalent popular remedies were used. The profuse use of papaya and banana was reportedly used to induce abortions. Women also reported using herbal decoctions or “heat-inducing foods” to terminate the pregnancy. Along with fruits, oils and alcohol were also believed to induce abortion. Hospital was always the last resort for various reasons narrated earlier.

*“Eat a raw banana or raw papaya. Or drink eucalyptus water or oil. Some people would eat jackfruit if it did not get aborted, because it [/jackfruit/] has heat. Even then if it does not get aborted; they have a hospital as the last option.”* (IDI_U_03, privately operating FSW, urban).

Besides fruits, use of alcohol is another method of inducing abortion:

*“In my house, my friend got this [/tablet for abortion/] two times. For it, you need alcohol [/along with this tablet/]. You have to drink raw alcohol, don't add water in that, you get Rum, you drink that in 2–3 days and the womb becomes empty. The stomach becomes empty [/pregnancy termination/]; means after drinking raw alcohol [/without adding anything to it/], eat hot eggs, eat hot omelet, drink hot coffee, don't add sugar, drink very warm black coffee, drink thumbs up [/a brand of fizzy soda/]. In 2–3 days, it [stomach] will get emptied.”* (IDI_U_06, brothel-based FSW, urban).

Use of over-the-counter medications for abortion was commonly reported by FSWs from the urban site.

*“They take medicines if they want to have an abortion. Some people use other methods. You get medicine for that. It costs two thousand rupees…You get it in the medical shop, at some other places and can also get it here in the hospital, doctors also give it…You should ask for the pills for abortion at medical shop. They will give.”* (IDI_U_14, brothel-based FSW, urban).

“*What else? You get one pill at rupees 2,500, for dropping [/terminating pregnancy/]. You can abort till about two to two and half months, you can get that pill at the medical shop.”* (IDI_U_01, brothel-based FSW, urban).

Among rural FSWs in addition to fruits, use of spices such as turmeric and honey along with honey, or sugarcane juice was reportedly used for inducing abortion. Unregulated and rampant use of painkillers have been reported to be used by the FSWs in rural settings.

*“What do they do? What is that [/thinking/]? Black pepper is there, isn't it, that you should take. The black pepper powder. Add it in honey and eat that, with that…then there is pain…because of that it pains, it is emptied [/pregnancy is terminated/].”* (IDI_R_04, hidden FSW, rural).

“*Once a woman told me to drink water mixed with turmeric. I did it, but nothing happened to me. I drank 100–200 g of turmeric. Even then the problem [/pregnancy was not terminated/] was not solved and I didn't get periods.”* (IDI_R_10, street-based FSW, rural).

“*It is said that eating papaya, eating banana, in large quantities, not just one-two pieces, eating lot of bananas or papaya for 2–4 days consecutively, whenever they get time, they eat papaya, raw papaya…many women know that if the tablets meant for body ache etc. are consumed for many days consecutively; it can lead to abortion. They consume them for 4–4, 5–5 days continuously three times a day, it leads to an abortion. If it is an early pregnancy, diagnosed early, may be it [/abortion/] happens easily. Old women [/traditional community level experienced but not formally trained midwives/] in their group also have the training of the methods like giving pressure on the abdomen etc*.” (KII_08, Counselor).

### Differential facility preference

Sex workers at the rural and the urban study sites preferred government health facilities over private health facilities for deliveries. The major reason for preferring government facilities for delivery of unintended pregnancy was that it allowed them to give away the child.

*“Get proof. [/It is/] easy to give away [/the child/].”* (KII_07, NGO representative).

Although the FSWs prefer government health facilities for delivery, these women lacked the necessary legal documents to get incentives, as pointed out by a key informant:

*“Without the card [/proof for income/], the delivery will happen like delivery [/standard delivery with no government benefits/]. Must show that they have a yellow ration card for incentives.”* (KII_11, healthcare provider).

Moreover, the brothel owner also described the preference for home deliveries and not institutional delivery services.

*“Hit on their thigh while delivering [/institution/]. [/Because of/] fear, delivered at home…They go [/to the health facility/] only if needed later.”* (KII_17, brothel owner).

HIV positive pregnant FSWs were not encouraged at private facilities. Stigma and discrimination were hidden but still evident.

*“Delivery can be done there only [/Government facility/]. HIV cannot be treated in private…Everything is free of cost there for her. Tablets, medicines, for her baby, for her; their HIV testing also gets done. That's why they [/private facility people/] send them there.”* (KII_22, Outreach worker).

“*If a woman is HIV positive, the percentage of being delivered in private is very less.”* (KII_14, program personnel).

However, there were challenges—FSWs at the rural sites shared fear of being seen at the health facilities while being pregnant, and being identified by customers and neighbors. FSWs at the rural sites also reported experiencing discrimination by HCPs during pregnancy and shared experiences of being verbally abused and requiring spousal authorization for pregnancy related services.

*“When we visit hospital, we can't introduce ourselves as a sex worker. As a result, we cannot discuss our problems freely and even if we tell them our problem, pregnancy or abortion…they i.e., the doctor or worker there, they expect your partner to be with you, mostly at the time of abortion or during pregnancy. That time we can't take our partner there. Without partner they don't give any further treatment or they expect him to come.”* (IDI_R_10, street-based FSW, rural).

The FSWs at the urban sites felt more accepted at the government health facility for the pregnancy-related services.

Community collectivization and empowerment, and interventions by CBOs or NGOs seem to be an imperative for the most vulnerable group of sex workers residing in the rural and urban areas.

*“How should we go till there [/healthcare facilities/]? Then they need somebody. Now there is some NGO, after going to an NGO they need some person in whom they have faith. If I have to undergo an operation, so after going with some person in whom I [/FSWs/] have trust, that whatever is going to happen with me, it's going to be good. They generally do not go by themselves/ alone. Then friends–friends…if they are there, if one of them had some experience such as- O here the operation was done and I did not have a problem. This faith is sought. Woman who has come to the NGO and if she had good experience, she tells other girls. They have more trust on the NGO. All women come to know. Even if she is home based, they by themselves come to the NGO for help with their own problems.”* (KII_01, NGO representative).

A small example of collectivization was shared among rural sex workers.

*“One patient I had taken earlier, isn't it…how[/so/] many are working in our community…[/they/] fought with all [/for a service that was denied/]. [/We/] all went and stood there [/at a healthcare facility/]…This delivery has to happen here…the child was born…if cesarean, then also it should happen here, [/normal/] delivery needed, then also it should be done here only.”* (FGD respondent, rural).

Women with HIV infection also preferred government facilities for child delivery:

*“Convenience for HIV positive women since the doctor knows the HIV status. HIV, the doctor already knows of HIV, and so accordingly, they do her delivery.”* (IDI_U_03, privately operating FSW, urban).

While the FSWs generally shared the knowledge that the child is at the risk of acquiring HIV at the time of birth or breastfeeding, in all practical senses, they did not mention early detection of pregnancy, early and timely antenatal care and HIV testing to prevent HIV transmission. A sex worker shared that one of her two children is HIV negative because she was given “*the tablet”:*

*“I found out about my HIV in the beginning in* = *year* =*. Even my child has it, but at present one of my other children is there, isn't it? That child does not have. We did all these tests in private, that child does not have [/HIV/], during my infected child's time, I did not take medicine. At the time of my uninfected child, I was given the tablet [/ART/], that child does not have.”* (IDI_2, FSW).

## Discussion

The RMNCH+A program seemed to fail in addressing FSWs' issues of unintended pregnancy. The RSH services among FSWs were sub-optimally utilized due to (I) not being able to negotiate to prevent pregnancy, (II) not using non-barrier or dual methods of contraceptives owing to complacency, myths and misconceptions, (III) patriarchal attitude of program as a barrier to access and utilize contraceptive services, and (IV) stigma—a barrier to accessing contraception and safe pregnancy termination services. Use of male condoms for pregnancy prevention emerged as a priority over HIV prevention.

The factors related to FSWs' unmet RSH needs and their engagement in RSH care can be categorized under the social ecological model (40) as intrapersonal, interpersotrust, that whatevernal, institutional, community and policy level factors. At the policy level, the PPTCT program of the Government of India emerged as an enabler of institutional deliveries among HIV infected FSWs. The RSH and family planning needs coexist for all women but the two programs run parallel and do not converge to address FSWs' needs. We recommend integrating the two programs to address FSWs' RSH needs. The common intervention across the national HIV Targeted Intervention program and the RMNCH+A program is promoting condom use, condom promotion in HIV targeted intervention focuses on preventing HIV among this population, and prevention of unintended pregnancy is not a primary concern. Experiences of frequent condomless sex and unintended pregnancy were commonly reported by FSWs in this study. The estimates of unintended pregnancy among FSWs in India are not available. An estimated 70 unintended pregnancies per 1000 women aged 15–49 years in India have been reported by Singh et al. (41). To extrapolate from this report, unintended pregnancies would be very high among the FSW population in India, as reported the world over (42).

At the individual level, a high level of knowledge about HIV prevention by using condoms emerged, but in-depth exploration showed the practice gap. Reliance on condom-only as a contraceptive method by the FSWs in this study has also been reported by FSWs from other parts of the country (16). However, the condom failures were the predominant events in FSWs' lives in this study and other studies as well (15, 24, 43). The consequent unintended pregnancies were frequent among the FSWs in this study, as reported by other researchers (6, 20, 44–46). The results are serious complications associated with pregnancy termination (10). Therefore, the uptake of non-barrier methods of contraception needs to be scaled up for the FSW population.

From the community and interpersonal level perspective, concerns about pregnancy were strong among FSWs. In alignment with other reports (47), stigma, both internalized and experienced over pregnancy, emerged strongly in our study. As FSWs reported in Tanzania (48), our study participants were afraid of losing clients if they became pregnant. There were psychosocial reasons for not using condoms. In an attempt to make their relationship more akin to marital relationships, FSWs did not use condoms as proof of fidelity to their partner. This is in contrast to the Kenyan study in which the women preferred condoms for themselves to get dual protection against pregnancy and STIs (15). The intentional tearing/ failure of condoms strongly suggests the need for strong promotion of dual methods of contraception among FSWs to prevent HIV infection as well as pregnancy among FSWs. At the same level of expenditure, increasing contraceptive use through family planning services and outreach among those not using contraceptives and not wanting to get pregnant averts almost 30% more HIV-positive births than HIV counseling and testing coupled with ART (49).

Non-barrier methods of contraception were also found not acceptable to women owing to several myths and misconceptions that were similar to the reports from women in Ghana (50), Cambodia (51), and South Africa (52). Therefore, an effective Information Education Communication (IEC) that the condom/ barrier method is an agency for triple prevention of (I) STI, (II) HIV, and (III) pregnancy is recommended. It is critical that the family planning program in India develop an IEC pertaining to non-barrier methods of contraception to address the myths and misconceptions. Simple infographics and body maps delivered through social/ print media, explaining the placement of non-barrier contraceptives inside a woman's body could increase acceptance and use of non-barrier methods of contraception. Behavioral disinhibition in the context of protection from STI and HIV was observed. When condoms were used for pregnancy prevention, FSWs tended to forgo condom use when they were using methods other than condoms. Therefore, the prevention of unintended pregnancy emerged to be a greater motivator and a priority for using condoms compared to HIV prevention. This overdependence on condoms as contraception culminates in risk for pregnancy, as discussed earlier.

There is an immediate opportunity to explore how pregnancy prevention, pregnancy testing services and elements of reproductive health services such as urine pregnancy testing can be integrated within existing HIV Targeted Intervention programs. Urine Pregnancy Tests kits could be made available at the Drop-in Center (DIC) for the HIV Targeted Intervention program. The peer educators could be trained to deliver the kits and interpret the results. The routine health checkup at the HIV Targeted Intervention sites may include a holistic approach to women's reproductive health rather than focusing on HIV and STIs alone. We recommend expanding the national condom promotion program and increasing the sales of condoms at the rural sites through non-traditional outlets and at stigma-free settings.

The institutional level barrier among FSWs in the study was the experience of discrimination from healthcare providers while accessing antenatal care and abortion services. The discrimination was more pronounced at the rural sites. We recommend medical training to the healthcare providers. We recommend sensitivity and law and enforcement training (53) to the healthcare providers to improve their knowledge about FSWs' needs, to deliver health services without prejudice and monitor services for FSWs. Community mobilization has been proven to be one of the structural interventions to improve the risk environment among vulnerable populations for HIV prevention (54, 55). Therefore, for FSWs to campaign for their reproductive rights, a similar approach can be adapted by bringing together FSWs through mobilization, participation, and empowerment processes, and providing them with the space and resources and an environment to act together. Policy or environmental changes that are beyond the FSWs control are also required to prevent unintended pregnancy and STIs. Value education and gender equity training may be imparted to children in young age and be woven into the social fabric to help them recognize the impact of gender stereotypes and address the inequalities that arise from them.

Similar to the experiences of women undergoing abortions in a legally restrictive context (56), the participants in our study felt constricted. Male involvement for RMNCH might be a successful policy for general women but not for FSWs. The pregnancy, pregnancy termination or permanent contraception service environment is patriarchal and, therefore, very restrictive for women who are not married and/ or into sex work, thus limiting their access to legal and safe services. The requirement of having a spouse's name or marital status or having borne at least one child for permanent contraception services (57) needs to be reformed and made optional information (58). Such reforms can become gender-friendly, especially for FSWs. In the case of FSWs, the PPTCT program's success may not only be based on linking HIV positive pregnant women to ART but also on the prevention of unintended pregnancies and access to safe abortion services. PPTCT services in India are directed toward women from the general population and not FSWs who do not reach the primary health facilities for antenatal care. Late pregnancy discovery is a missed opportunity for FSWs who could get linked to the Medical Termination of Pregnancy (MTP) and/ or early antenatal care. In the PPTCT program (59), among the four prongs of elimination of pediatric HIV, the program has historically focused more on preventing HIV (prong 1) among FSWs and preventing vertical transmission from the mother to the child through ART (prong 3) (60). While these interventions represent major public health achievements, the current impact of the PPTCT programs is limited by their challenge to effectively link with sexual and reproductive services and address the contraceptive needs of women living with or at risk of HIV (prong 2) (61–65). Understanding FSWs' access to reproductive and sexual health services and their reproductive health needs may inform the program to contribute to the 95–95–95 global goals of HIV elimination by 2030 (66). The future availability of a broader range of Multipurpose Prevention Technologies (MPTs) will be crucial to the shared prevention toolbox. It may also be an opportunity to further integrate family planning and HIV programs.

This is a qualitative study, and the results would not be generalizable to other contexts and populations beyond FSWs within similar settings and health systems. Although efforts were taken to build rapport and trust with the community and the participants, there could likely be a social desirability response bias in answering the sensitive questions.

## Conclusion

Sexual violence and coercive sex increase an FSW's vulnerability to unintended pregnancy, which she wants to avoid. While condoms are the preferred primary method of contraception yet, prevalence of condom failure is high owing to violence, economic and emotional needs of sex workers, alcohol intoxication and coercive sex. Pregnancy prevention is a greater motivation for condom use than HIV prevention among FSWs. Therefore, it is time to relook at their RSH needs, especially in the prevention of unintended pregnancy and redirect the public health resources that have gone into other intervention programs for FSWs. Knowledge of use of condoms for HIV prevention exist but in practice, FSWs primarily use them for contraception. Information Education Communication (IEC) should strongly promote condoms/ barrier method for triple prevention of (I) STI, (II) HIV, and (III) pregnancy.

Since “reproductive” health is a woman's issue, rethinking is required on the present documentary requirements of marriage and spouse's details and consent. Equal protection of laws against rape and other forms of violence is crucial for FSW to protect their reproductive and sexual health. Facilities need to earmark space and ambient environment for FSWs to access contraceptive, antenatal care, safe pregnancy termination and delivery services. Stigma reduction at HCP's level is essential.

## Data availability statement

The original contributions presented in the study are included in the data. Further inquiries can be directed to the corresponding author.

## Ethics statement

The studies involving human participants were reviewed and approved by ICMR-National AIDS Research Institute. The patients/ participants provided their written informed consent to participate in this study.

## Author contributions

SSh: conception and design, methodology, data collection, data analysis and interpretation, drafting of the manuscript, making critical revisions to the manuscript, and final approval for the completed version. SSa: conception and design, methodology, data analysis and interpretation, supervision, making critical revisions to the manuscript, and final approval for the completed version. All authors contributed to the article and approved the submitted version.
